# Antioxidant and Hepatotoxicity Ameliorative Potential of *Mentha canadensis* Leaves Against Paracetamol‐Induced Hepatic Damage in Mice

**DOI:** 10.1002/fsn3.71279

**Published:** 2025-11-28

**Authors:** Asma Ul Husna Biswas, Tasnima Kamal, Azmin Akter, Sharmin Akter, Azadur Rahman Bhuiyan, Zinnat Ara Moni, Abdul Auwal, Nitai Roy, Shakhawoat Hossain, Farhadul Islam

**Affiliations:** ^1^ Department of Biochemistry and Molecular Biology University of Rajshahi Rajshahi Bangladesh; ^2^ Department of Biochemistry and Molecular Biology Patuakhali Science and Technology University Patuakhali Bangladesh; ^3^ School of Medicine and Dentistry Griffith University Gold Coast Queensland Australia

**Keywords:** ABTS, antioxidant, DPPH, glutathione, hepatotoxicity, *Mentha canadensis*, paracetamol

## Abstract

*Mentha canadensis* (wild Mint), a member of the Lamiaceae family, is traditionally used in herbal medicine, teas, and as a food additive in various regions, including Bangladesh. This research is designed to evaluate the hepatoprotective activity of *M. canadensis* leaf methanolic extract (MCLME) along with its antioxidant and anti‐inflammatory potential. The in vitro antioxidant potential was evaluated through free radical scavenging assays such as ABTS and DPPH, as well as by a lipid peroxidation test. The anti‐inflammatory property of MCLME was examined using albumin denaturation and RBC membrane stabilization assays. Furthermore, qualitative and quantitative analyses of phytochemicals were carried out to investigate the basis of its therapeutic activity. A paracetamol‐induced hepatotoxicity Swiss‐albino mice model was used to assess the hepatoprotective activity of MCLME. Phytochemical analysis of MCLME confirmed the presence of alkaloids, flavonoids, triterpenoids, coumarins, saponins, and steroids, which may attribute to the extract's bioactivity. The extract also exhibited notable antioxidant and anti‐inflammatory activity. In a paracetamol‐induced hepatotoxicity mouse model, treatment with MCLME showed a significant (*p* < 0.0001) reduction of elevated serum glutamate pyruvate transaminase (SGPT), serum glutamic‐oxaloacetic transaminase (SGOT), serum alkaline phosphatase (ALP), total bilirubin, and pro‐inflammatory cytokine IL‐6. Also, histopathological analysis further confirmed the protective effects of MCLME on hepatic architecture. Additionally, MCLME restored the levels of hepatic glutathione (GSH), the key endogenous antioxidant, in treated mice. The hepatoprotective effects of the extract are comparable with the standard hepatoprotective agent, Silymarin (50 mg/kg body weight). Altogether, our findings indicate that *M. canadensis* demonstrates significant hepatoprotective, antioxidative, and anti‐inflammatory characteristics, potentially serving as a natural alternative therapeutic agent for liver injury.

## Graphical abstract

This study demonstrates the hepatoprotective potential of *M. canadensis* leaf methanolic extract (MCLME), through its antioxidant and anti‐inflammatory activities, against paracetamol‐induced liver injury in mice. MCLME significantly reduced serum liver biomarkers, restored glutathione and IL‐6 levels, and improved hepatic histology, indicating membrane stabilization and liver function recovery. In vitro assays further confirmed its strong antioxidant and membrane‐protective effects. Overall, MCLME effectively mitigates oxidative stress, inflammation, and hepatocellular damage, with efficacy comparable to the standard hepatoprotective agent, Silymarin.

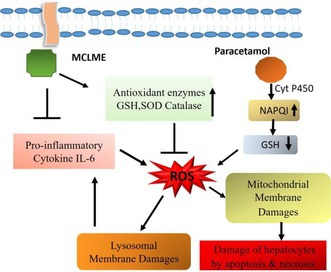

## 1 Introduction

Liver conditions are an important concern for healthcare around the world, contributing to high rates of morbidity and mortality. Drug‐induced liver injury (DILI) is one of the major problems, posing considerable challenges in clinical and regulatory settings (Li et al. 2023). Drug‐induced hepatotoxicity manifests in various forms, from fulminant hepatic failure to asymptomatic increases in liver enzymes (Parmar et al. 2010). A noteworthy example is paracetamol (acetaminophen), the most extensively used analgesic and antipyretic worldwide, including in Bangladesh (Davies et al. 2024). While safe at therapeutic doses (< 4 g/day in adults, or < 60–75 mg/kg/day in children), acute liver damage (ALI) and possibly catastrophic liver failure can result from excessive consumption.

Previous research has employed paracetamol to cause liver damage in mice in order to assess the hepatoprotective properties of chemical or pharmacological components. (Zakaria et al. 2020; Mahmood et al. 2014). Its toxic metabolite, NAPQI, is produced by cytochrome P450 enzymes. Under normal conditions, NAPQI is rapidly detoxified by glutathione (GSH) and excreted. In overdose conditions, excessive NAPQI is produced, exceeding the liver's GSH stores. Depleted GSH leads to the accumulation of NAPQI, which binds covalently to hepatic proteins, causing oxidative stress and mitochondrial dysfunction. Generation of reactive oxygen species (ROS) triggers cell death via necrosis and apoptosis, leading to hepatic inflammation and tissue damage (Liao et al. 2023; García‐Román and Francés 2020). Although N‐acetylcysteine (NAC) is the standard therapeutic for paracetamol toxicity (Licata et al. 2022), its short half‐life necessitates frequent dosing, which can cause adverse effects with prolonged use (Hamedinasab et al. 2023). This drawback emphasizes the urgent need for safer, more effective, and long‐lasting therapeutic alternatives that can improve patient outcomes and mitigate the risks associated with hepatotoxicity.

The use of phytochemicals offers a promising approach in modern medicine for managing liver toxicity. These plant‐based compounds contain a wide range of bioactive ingredients, such as flavonoids, polyphenols, and saponins, which have been demonstrated to possess significant hepatoprotective properties (Karim et al. 2020). These phytochemicals demonstrate antioxidant, anti‐inflammatory, and hepatoprotective properties, rendering them viable choices for mitigating liver damage induced by toxins and pharmaceuticals (Kanchana and Sadiq 2011; Mahesh et al. 2010). Moreover, phytochemicals generally exhibit fewer adverse effects compared to synthetic medications, enhancing their appropriateness for long‐term liver health maintenance. Integrating phytochemicals into liver toxicity treatment, whether through herbal formulations, dietary supplements, or plant‐derived therapies, could provide a safer and more holistic therapeutic alternative (Rani et al. 2024). However, further rigorous preclinical and clinical research is essential to establish their efficacy, safety, and optimal dosing before widespread clinical implementation.

This study employed *Mentha canadensis* L., commonly known as Canadian mint or wild mint, a perennial species of the Lamiaceae family with a broad geographical distribution across North America and parts of Asia, particularly in China and Japan (Salehi et al. 2018). Recognized for its rich phytochemical profile, this species has been extensively used in traditional medicine systems (Kumar and Dudhe 2014), with documented applications in treating multiple ailments, including respiratory disorders (colds, coughs, asthma), gastrointestinal complaints (indigestion), inflammatory conditions, and febrile illnesses (Saqib et al. 2022). Studies suggest that *M. canadensis* shows broad‐spectrum antimicrobial effects against bacterial and fungal pathogens (Krivonogova et al. 2021). Its compounds have been shown to have antiviral activity against Omicron BA.1 (Chou et al. 2023). This species contains a high number of flavonoids and phenolic acids that exhibit strong antioxidants and anti‐inflammatory properties and help neutralize free radicals, reducing oxidative stress‐related diseases (Li et al. 2017). Mint tea, especially peppermint tea, has traditionally been used to ease gastrointestinal discomfort and upper respiratory issues. Its menthol content possesses antispasmodic properties that help relax gastrointestinal muscles, potentially relieving stomach pain and bloating (Ćavar Zeljković et al. 2021). Although the bioactive lead compounds of *M. canadensis* have been previously investigated for their broad range of pharmacological effects, the potential of its methanolic leaf extract in mitigating liver toxicity remains unexplored. Given the limitations of current treatments and the promising pharmacological properties of *M. canadensis* , this study attempted to examine the possible hepatoprotective benefits of methanolic leaf extract against paracetamol‐induced hepatic injury in Swiss albino mice. This research may aid in the development of innovative therapeutic techniques for drug‐induced hepatotoxicity by investigating its ability to mitigate oxidative stress and inflammation.

## 2 Materials and Methods

### 2.1 *Mentha canadensis* Leaves Methanolic Extract (MCLME) Preparation

During the spring (March 2024), mature leaves of *M. canadensis* were collected from a local region in Rajshahi, Bangladesh, and authenticated by taxonomist Dr. A.H.M. Mahbubur Rahman (voucher no. LF130) at the Department of Botany, University of Rajshahi. Following meticulous cleansing with tap water, the fresh leaves were permitted to air dry in the shade at ambient temperature before being pulverized into a fine powder using a mechanical grinder. The powder was contained in an airtight zipper bag. Methanol was used as the solvent for the extraction process because it can dissolve polar and certain relatively nonpolar molecules, making it suitable for extracting a wide range of components. Using a magnetic stirrer, 150 g of powdered leaves were incorporated with 500 mL of methanol and constantly agitated for 24 h at ambient temperature (25°C). After that, Whatman No. 1 filter paper was used to filter the mixture to get an extract free of particles. Double cold maceration was applied to the residue. To concentrate the combined extracts, a rotary vacuum evaporator (40°C and 120 millibar) was used (Hahnshin Scientific, Korea) and stored in a refrigerator for subsequent analysis.

### 2.2 Test Animals

Adult male ICR mice (6–8 weeks old; 25 ± 5 g body weight) were obtained from the animal facility of the Department of Biochemistry and Molecular Biology, University of Rajshahi. The mice were kept in clean, well‐ventilated rooms in standard polypropylene cages with a 12‐h light/12‐h dark cycle. Following a natural day‐night cycle, standard food and drinking water were provided on the basis of need.

### 2.3 Ethical Clearance

The Institutional Animal, Medical Ethics, Biosafety, and Biosecurity Committee (IAMEBBC) at the Institute of Biological Sciences, University of Rajshahi, Bangladesh (128/320‐IAMEBBC/IBSc), sanctioned the utilization of animals for this research. In addition, the work described herein was carried out following the National Institutes of Health Office of Laboratory Animal Welfare policies and laws and complied with the ARRIVE guidelines. Isoflurane anesthesia was performed for all surgeries, and all efforts were made to lessen the suffering of the animals.

### 2.4 Experimental Design

Twenty‐four mice were separated into 4 groups (*n* = 6 per group). The hepatoprotective potential of MCLME was evaluated over 5 days using a paracetamol‐induced hepatotoxicity model as described by Karim et al. (2020). Controlled conditions (25°C ± 2°C) were maintained for the mice under natural light and dark cycles during the experiment. Paracetamol (Sigma Aldrich, St. Louis, Missouri, USA) was administered at 500 mg/kg body weight/day via oral route to induce hepatic injury to mimic overdose‐induced liver injury in humans, as paracetamol overdoses are well established for acute liver failure in humans. A 500 mg/kg body weight is a standardized high dose for controlled hepatotoxicity in mice used in many studies (Mahmood et al. 2014). The subsequent treatment plan was designed in this study: Group 1 (Normal control): Administered distilled water orally for 5 days. Group 2 (Pathological control): Administered paracetamol (500 mg/kg body weight/day, orally) for 5 days. Group 3 (Standard control): Administered silymarin (50 mg/kg body weight/day, orally) (Sigma Aldrich, St. Louis, Missouri, USA) following paracetamol (500 mg/kg body weight/day, orally) for 5 days. Group 4 (Test group): Administered MCLME (200 mg/kg body weight/day, orally) following paracetamol (500 mg/kg body weight/day, orally) for 5 days.

### 2.5 Collection of Blood and Preparation of Liver Samples

Mice were anesthetized with isoflurane and euthanized 24 h after the final treatment. Blood was extracted from the thoracic artery, permitted to coagulate, and subjected to centrifugation at 3500 rpm for 15 min at 4°C to isolate serum, which was thereafter kept at −20°C. Following the liver tissues' excision, they were cleaned with phosphate‐buffered saline (PBS), labeled, weighed, and homogenized in 0.1 M phosphate buffer (pH 7.0) with 0.5% Triton X‐100 to create a 5% liver homogenate. Finally, the homogenates of liver were centrifuged at 3000 rpm for 15 min at 4°C, and the supernatants were utilized for biochemical assay (Sanjeev et al. 2019; Kabir et al. 2024).

### 2.6 Phytochemical Screening of MCLME

#### 2.6.1 Qualitative Analysis

##### 2.6.1.1 Test for Alkaloids

(a). Hager's test: The solvent‐free extract was stirred with diluted hydrochloric acid, filtered, and a few milliliters of filtrate were mixed with Hager's reagent along the side of a test tube. The presence of alkaloids was confirmed by a bright yellow precipitate. (b). Wagner's test: The extract was dissolved in diluted hydrochloric acid, filtered, and treated with Wagner's reagent (iodine in potassium iodide). A brown or reddish precipitate indicated the presence of alkaloids. (c). Dragendroff's test: Dragendroff's reagent, a solution consisting of potassium bismuth iodide, was applied to the filtrate. The emergence of a crimson precipitate confirmed the presence of alkaloids.

##### 2.6.1.2 Test for Flavonoids

The alkaline reagent assay was used to detect flavonoids. Two milliliters of the sample extract were combined with a few drops of sodium hydroxide solution. A bright yellow color appeared, which turned colorless upon the addition of diluted hydrochloric acid, indicating flavonoids.

##### 2.6.1.3 Test for Carbohydrates

(a). Molisch's test: Two milliliters of the plant extract were mixed with Molisch's reagent and carefully layered with concentrated sulfuric acid. A reddish violet ring at the interface confirmed the presence of carbohydrates. (b). Fehling's test: After adding equal volumes of Fehling's solutions A and B to two milliliters of extract, the mixture was heated for 10 min in a water bath. Reducing sugars were indicated by a brick‐red precipitate.

##### 2.6.1.4 Test for Steroids (Libermann‐Burchard's Test)

1 mL of acetic anhydride was combined with 1 mL of 5% cholesterol that had been dissolved in chloroform. The same tube is then filled with two drops of H_2_SO_4_ and thoroughly shaken. Steroids were detected by gradual changes in color from pink to deep green.

##### 2.6.1.5 Test for Triterpenoids (Salkowski Test)

1 mL of chloroform was used to dissolve 10 mg of the extract after the addition of 1 mL of concentrated sulfuric acid. A reddish‐brown coloration at the interface indicated triterpenoids.

##### 2.6.1.6 Test for Coumarins

A small volume of 2 N sodium hydroxide solution was introduced to the extract. The emergence of a dark yellow hue indicated the presence of coumarins.

##### 2.6.1.7 Test for Saponins (Foam Test)

Equal volumes (5 mL) of the extract and distilled water were shaken vigorously and heated. Stable foam formation confirmed the presence of saponins.

#### 2.6.2 Quantitative Analysis of Phytochemicals

##### 2.6.2.1 Determination of Total Flavonoids Content

The aluminum chloride colorimetric method was used to determine the total flavonoids content, with catechin as the standard. Briefly, 0.5 mL of MCLME was mixed with 0.15 mL of 5% NaNO_2_ and 0.3 mL of 10% AlC1_3_.6H_2_O. Following the incubation process 1.0 mL of 1 M NaOH was introduced, and absorbance was measured at 430 nm. The total flavonoid content was expressed in catechin equivalents per gram of dried sample (mg CA/g), derived from the calibration curve (standard curve equation: *y* = 0.0033*x* + 0.0107, *R* ^2^ = 0.9999) (Shraim et al. 2021).

##### 2.6.2.2 Determination of Total Phenolic Content

Total phenolic content of MCLME was assessed using the Folin–Ciocalteu reagent (FCR) method with gallic acid as the standard. 0.3 mL of various MCLME concentrations was mixed with 2.25 mL of sodium carbonate (6%) solution and 2.25 mL of Folin–Ciocalteu reagent. Absorbance was measured at 760 nm. Total phenolic contents were expressed in terms of Gallic acid equivalent, GAE (standard curve equation: *y* = 0.0085 + 0.0357, *R* ^2^ = 0.9996) mg of GAE/g of dry extract (Blainski et al. 2013).

##### 2.6.2.3 Determination of Total Flavonol Content

Total flavonol content of MCLME was quantified utilizing Kumaran and Karunakaran (2007) method. In brief, a mixture of 0.5 mL MCLME, 2.0 mL of 2% AICI_3_, and 3.0 mL (50 g/L) sodium acetate solution was made, which was incubated for 2.5 h at 20°C, and absorbance was measured at 440 nm. The total flavonol content was quantified as quercetin equivalents (QU) using the standard curve equation *y* = 0.0066*x*—0.0384 (*R* ^2^ = 0.9998). Results are presented as quercetin equivalents per gram of the dried sample (mg CA/g) (Kumaran and Karunakaran 2007).

### 2.7 Determination of Antioxidant Activity by the 2,2‐Diphenyl‐1‐Picrylhydrazyl (DPPH) Radical Scavenging Method

Using a slightly altered version of the Brand‐Williams et al. (1995) approach, the 2, 2‐diphenyl‐1‐picryl‐hydrazyl (Sigma‐Aldrich) radical was used to examine MCLME's capacity to scavenge free radicals. In summary, 3 mL DPPH solution (0.1 mM) prepared in methanol was mixed with 1 mL of the methanolic extract solution or ascorbic acid, which served as the standard. The reference control was ascorbic acid. The mixture was thoroughly vortexed and underwent incubation in the dark at room temperature for 30 min. The absorbance of the resultant solution was measured at 517 nm utilizing a UV spectrophotometer.

### 2.8 Determination of Antioxidant Activity by ABTS Free Radical Scavenging Assay

The ABTS radical scavenging potential of MCLME was assessed with minor adjustments to the methodology established by Re et al. (1999). To generate ABTS radical cations, 2.45 mM potassium persulfate (1/1, v/v) was introduced to a 7 mM ABTS stock solution, immediately followed by incubation for 4–16 h until a stable absorbance was reached. The ABTS solution was subsequently diluted with water to achieve an absorption value of 0.70 ± 0.02 at 734 nm. Antioxidant activity was assessed by mixing 3.0 mL of ABTS+ solution with 1 mL of the sample at various concentrations. Ascorbic acid served as a positive control.

### 2.9 Lipid Peroxidation (TBARS) Assay

For this experiment, the procedure was adopted as proposed by Gutteridge and Halliwell (1990), with a little amendment. The lipid origin (10% liver homogenate), MCLME (1 mg/mL), and ascorbic acid (1 mg/mL) as standard at different concentrations were combined in this assay. In place of MCLME and ascorbic acid, methanol was used as a control. To continue lipo‐oxidation to the above reaction mixture, 10 mM FeCl_3_ was added, followed by incubation at 37°C for 1 h. Subsequently, TCA and TBA solutions were added to the mixture, and the resultant mixture was heat‐treated for 10 min at 100°C, accompanied by cooling and centrifugation at 3000 rpm for 10 min. Lastly, the absorbance of the pink colored supernatant formed was recorded at 532 nm.

The estimation of the percent inhibition of lipid peroxidation was based on the formula below:

$$\%\text{of inhibition}=\left(\left({\mathrm{OD}}_{C}-{\mathrm{OD}}_{S}\right)/{\mathrm{OD}}_{C}\right)\times 100$$

where OD_C_ = Absorbance of control solution. OD_S_ = Absorbance of the sample solution.

### 2.10 Anti‐Inflammatory Activity of MCLME by Inhibition of Protein Denaturation Assay

The albumin denaturation assay serves as an important preclinical method for screening compounds with possible anti‐inflammatory effects by evaluating their capacity to inhibit protein denaturation, which is a critical event in the inflammatory response. For this assay, 2 mL of different concentrations of MCLME was mixed with 2.8 mL of phosphate buffer solution (PBS, pH 6.4) and 0.2 mL of egg albumin obtained from a fresh hen egg. Then incubated at 37°C for 30 min, followed by heating at 70°C for 5 min. Test tubes were cooled, and absorbance was measured at 660 nm using a UV spectrophotometer. In this experiment, diclofenac‐sodium was used as a standard, and in place of the sample/standard, methanol was used as a control and treated similarly to determine absorbance (Sen et al. 2015).

### 2.11 Anti‐Inflammatory Activity of MCLME by Human RBC (hRBC) Membrane Stabilization Assay

The human RBC membrane stabilization (HRBC) assay is a well‐established method for assessing the anti‐inflammatory potential of a test sample. A 5 mL blood sample from a nonsmoking healthy human volunteer was collected for the examination of the in vitro anti‐inflammatory action of MCLME HRBC membrane stabilization assay. Sodium oxalate was used to prevent clotting. Blood was centrifuged at 3000 rpm for 5 min, and packed cells were washed 3 times with isosmotic saline (0.85%, pH 7.2) to remove cell debris. Then, a 10% (v/v) erythrocyte suspension was prepared for this experiment using isosaline. Stock RBC suspension (500 μL) was mixed with 5 mL of hypotonic solution (0.23%) containing MCLME at concentrations of 50, 100, 200, 400, and 800 μg/mL, while the control sample was mixed with a drug‐free solution. After incubating for 15 min at room temperature, the whole mixture was centrifuged at 3500 rpm for 15 min. Then, the absorbance of the supernatant was assessed using a UV‐spectrophotometer at 540 nm (Yesmin et al. 2020). Acetyl salicylic acid (ASA) was used as a reference standard.

### 2.12 Estimation of Glutathione (GSH) in Liver Tissue

GSH levels were quantified from the supernatant of 5% liver tissue homogenate, using the method described by Ellman (1959).

### 2.13 Biochemical Assay of Serum Liver Enzymes

The activity of the corresponding enzymes in serum was examined using SGOT, SGPT, ALP, and bilirubin activity assay kits (Human Diagnostics, Germany) following the manufacturer's procedure guidelines using an analyzer (Humalyzer‐3000, Germany).

### 2.14 Measurement of Inflammatory Cytokine IL‐6 in Serum

ELISA kits (Elabscience, USA) were used to measure IL‐6 proteins in serum following the manufacturer's guidelines.

### 2.15 Histopathology Study

Following cervical dislocation, liver tissues were collected from each group, and normal saline was used for washing. Samples were fixed in a solution of 10% neutral buffered formalin at 4°C for a duration of 24 h. The liver tissues were subsequently washed with xylene, embedded in paraffin at 56°C for 24 h using a hot air oven, and then sectioned to a thickness of 5 μm with a microtome. Before staining with hematoxylin and eosin (H&E), the sections underwent dehydration through an alcohol–xylene series. The samples were subsequently examined using a light microscope (Ibrahim et al. 2018).

### 2.16 Statistical Analysis

All experiments were conducted in triplicate, and the study results are presented as mean ± SEM (standard error of the mean). Data have been calculated by an unpaired t‐test using GraphPad Prism 8 software. A *p*‐value of < 0.05 was considered statistically significant.

## 3 Result

### 3.1 Phytochemical Profiling of *M. canadensis* Leaves

The phytochemical screening of *M. canadensis* leaf extract identified the presence of a wide range of bioactive compounds, including alkaloids, flavonoids, steroids, carbohydrates, and others, as summarized in Table 1. Positive results were obtained for alkaloids (Dragendorff's, Hager's, and Wagner's tests), carbohydrates (Molisch test), flavonoids, triterpenoids, coumarins, saponins, and steroids. Notably, Fehling's test for carbohydrates yielded a negative result. In addition, quantitative analysis indicated that the extract contained a high quantity of polyphenolic chemicals (Table 2).

**TABLE 1:** Qualitative test and phytochemical screening of bioactive compounds in *Mentha canadensis* leaves methanolic extract.

Test for compound	Name of the test	Presence of the compound
Alkaloids	Dragendorff's test	+
	Hager's test	+
	Wagner's test	+
Carbohydrates	Molisch test	+
	Fehling's test	−
Flavonoids	Alkaline reagent test	+
Triterpenoids	Salkowski test	+
Coumarins	Test for Coumarins	+
Saponins	Foam test	+
Steroids	Libermann‐burchard's test	+

**TABLE 2:** Composition of polyphenolic contents in *Mentha canadensis* leaves methanolic extract (MCLME).

Polyphenols content	Phenolic	Flavonoids	Flavonols
MCLME	71.68 ± 0.12 mg of GAE/g	133.11 ± 2.35 mg of CAE/g	89.703 ± 12.57 mg of QUE/g

*Note:* Data were expressed as mean ± SEM (standard error of the mean) (*n* = 3).

Abbreviations: CAE, catechin equivalent; GAE, gallic acid equivalent; QUE, quercetin equivalent.

### 3.2 Antioxidant Activity of MCLME

#### 3.2.1 DPPH Radical Scavenging Activity

**FIGURE 1:**
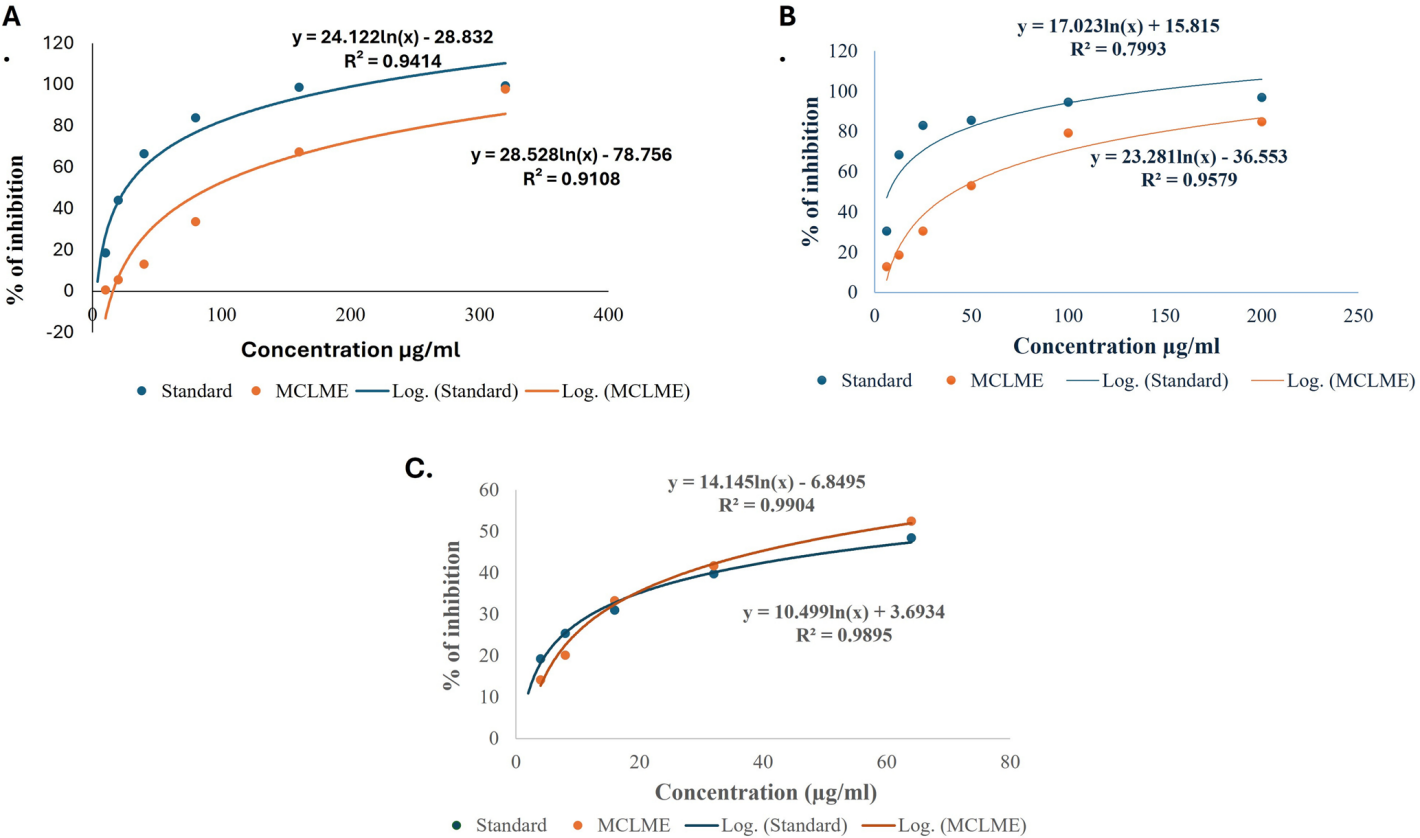
Antioxidant activity of *Mentha canadensis* leaves methanolic extract (MCLME). The extract exhibited potent free radical scavenging activity both in DPPH (A) and ABTS (B) assays with the IC_50_ values of 42.05 and 91.35 μg/mL, respectively. In the lipid peroxidation test, MCLME exhibited considerable inhibition of lipid peroxidation with the IC_50_ value of 55.61 μg/mL (C).

The antioxidant potential of *M. canadensis* leaves methanolic extract (MCLME) increased in a dose‐dependent manner, with a strong logarithmic correlation with concentration (*R* ^2^ = 0.9579). MCLME showed significant radical scavenging capacity, while the conventional antioxidant had stronger inhibition across all concentrations (*R* ^2^ = 0.7993). The standard's IC_50_ value was around 7.44 μg/mL, while MCLME's was 42.05 μg/mL, showing moderate but strong antioxidant effectiveness (Figure 1A).

#### 3.2.2 ABTS Radical Scavenging Activity

*Mentha canadensis* leaves methanolic extract (MCLME) demonstrated significant free radical scavenging activity, which was evaluated using the ABTS assay. The addition of the sample reduces the ABTS^•+^ radical, resulting in a noticeable decrease in color intensity. Antioxidant ability was assessed using the IC_50_ value, which indicates the concentration needed to inhibit 50% of ABTS^•+^ radicals. MCLME showed considerable antioxidant capacity with an IC_50_ value of 91.35 μg/mL, compared to the conventional antioxidant, ascorbic acid, which had a substantially lower IC_50_ value of 26.26 μg/mL (Figure 1B).

#### 3.2.3 Lipid Peroxidation Activity

In the lipid peroxidation assay, the IC_50_ values of MCLME and ascorbic acid were 55.61 and 82.26 μg/mL, respectively. The lower IC_50_ of MCLME indicates superior antioxidant efficacy, highlighting its potential as a natural source of antioxidants (Figure 1C).

### 3.3 Anti‐Inflammatory Activity of *M. canadensis* Leaves Methanolic Extract (MCLME)

#### 3.3.1 Inhibition of Protein Denaturation Assay

**FIGURE 2:**
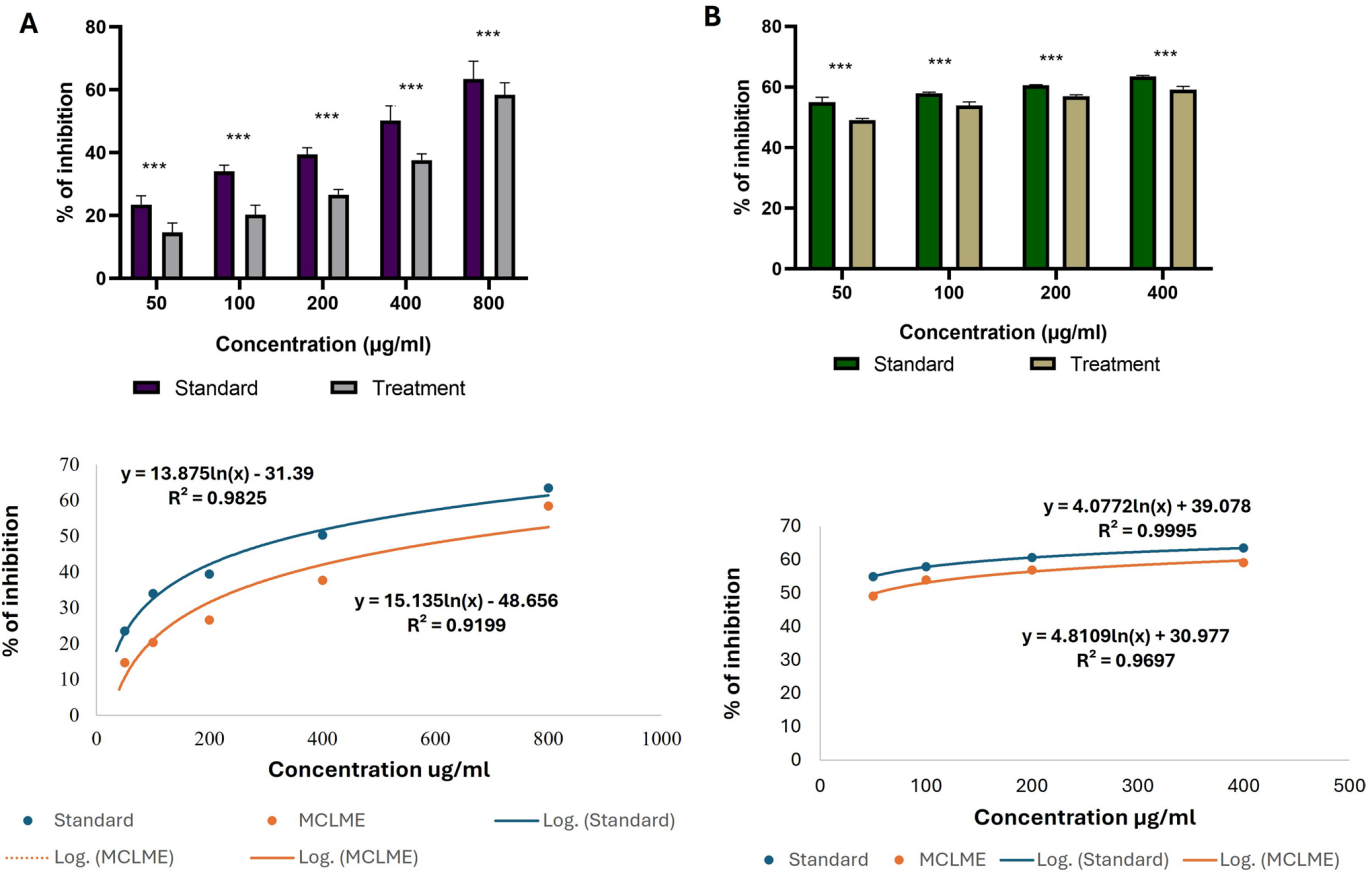
Anti‐inflammatory activity of *Mentha canadensis* leaves methanolic extract (MCLME). (A) In the albumin denaturation assay, MCLME demonstrated a notable inhibitory effect of 58.44%, closely similar to the inhibitory effect of the standard (63.45%). (B) In the human RBC membrane stabilization assay, MCLME manifested a noteworthy result with 59.19% inhibition of hemolysis of RBC membrane at a dosage of 400 μg/mL. Statistical significance is indicated as *p < 0.05, **p < 0.01, and ***p < 0.001 compared to the standard (acetylsalicylic acid) group.

In the present study, acetylsalicylic acid, used as the standard reference, exhibited the highest inhibition of protein denaturation (63.45%) at 800 μg/mL. At the same concentration, MCLME demonstrated a comparable inhibitory effect (58.44%), indicating its potential to effectively prevent protein denaturation and thereby exert significant anti‐inflammatory activity (Figure 2A).

#### 3.3.2 Human RBC Membrane Stabilizing Capacity

In this study, MCLME exhibited remarkable activity, achieving 59.19% inhibition of RBC membrane hemolysis at the highest tested dose (400 μg/mL). This effect was comparable to the standard drug, which showed a maximum inhibition of 63.50%, indicating that MCLME possesses strong anti‐inflammatory properties (Figure 2B).

### 3.4 Evaluation of GSH Levels

**FIGURE 3:**
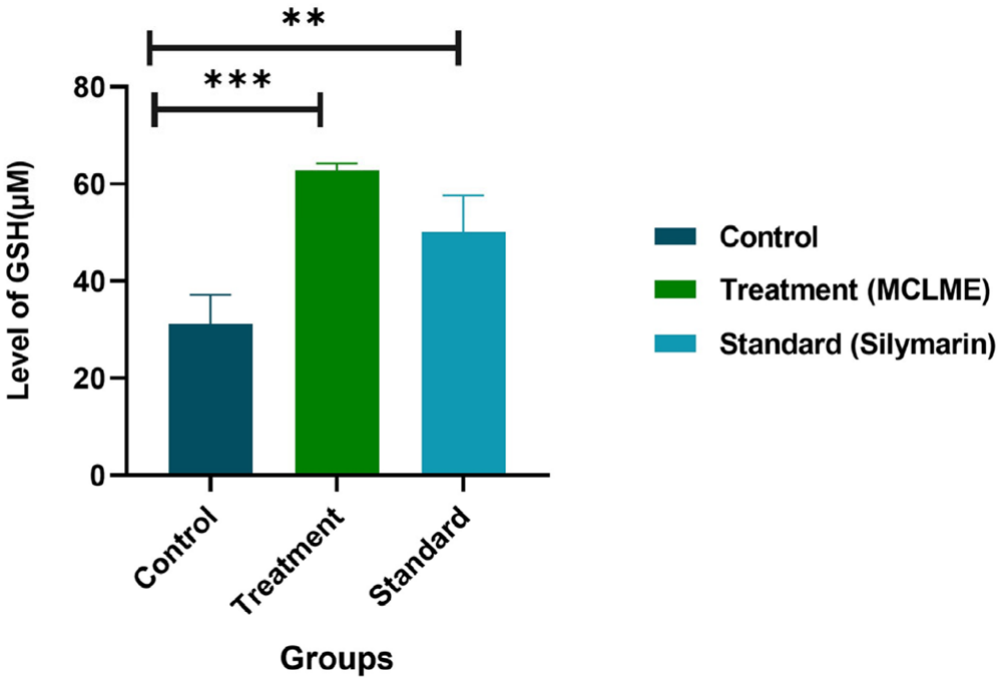
Effect of *Mentha canadensis* leaves methanolic extract (MCLME) on GSH activity in experimental animals. Notable increase of GSH level was observed in the MCLME‐treated group and silymarin‐treated group, while there was a decline of GSH level in the paracetamol‐treated group. Both the treatment and standard groups exhibited a significant difference when compared to the control group (paracetamol). Values are expressed as mean ± SEM (*n* = 6 per group). Statistical significance is indicated as **p < 0.01, and ***p < 0.001 compared to the paracetamol control group.

The quantity of glutathione (GSH) in liver homogenates was used to measure the hepatoprotective and antioxidant properties of MCLME. In comparison to the paracetamol‐induced control group (31.18 ± 5.48 μM), the GSH levels of both the MCLME‐treated group (62.82 ± 1.29 μM) and the standard group, treated with Silymarin (50.10 ± 6.87 μM), were significantly elevated, as illustrated in Figure 3. Silymarin was used as a standard antioxidant agent to compare the effect of MCLME on GSH level enhancement in experimental animals.

### 3.5 Assessment of Biochemical Parameters

**FIGURE 4:**
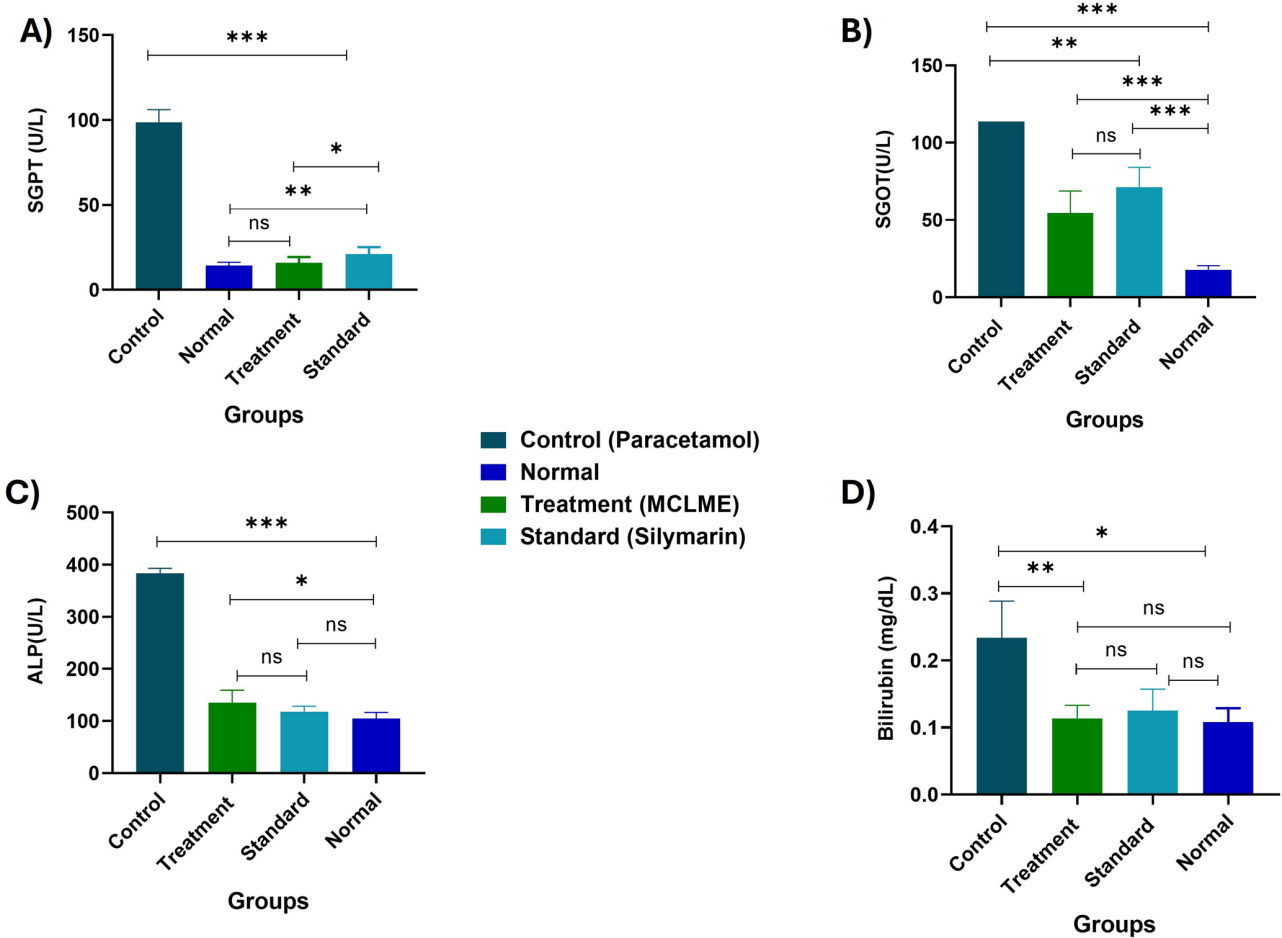
Effect of *Mentha canadensis* leaves methanolic extract (MCLME) on serum biochemical parameters in paracetamol‐induced hepatotoxicity on the 6th day. MCLME and standard group demonstrated the restoration of the liver biochemical parameters SGOT, SGPT, ALP, and bilirubin levels towards normal levels, while these parameters were deteriorating in the control group. (A) Effects of the extract on SGPT of experimental animals, (B) Effects of the extract on SGOT of experimental animals, (C) Effects of the extract on ALP of experimental animals, (D) Effects of the extract on bilirubin of experimental animals. Statistical analysis revealed that the control group (paracetamol) was significantly different from the treatment, standard, and normal groups. While non‐significant differences were seen between treatment and normal groups in SGPT, ALP, and bilirubin levels. Also, treatment and standard groups had seen statistically non‐significant SGOT test results. Values are expressed as mean ± SEM (*n* = 6 per group). Statistical significance is indicated as *p < 0.05, **p < 0.01, and ***p < 0.001 compared to the paracetamol control group.

The hepatoprotective effects of MCLME were evaluated by measuring crucial hepatic function biomarkers in mice subjected to paracetamol intoxication. As demonstrated in Figure 4, the paracetamol‐treated group had a significant increase in serum SGOT (113.63 U/L), SGPT (98.55 U/L), ALP (383 IU/L), and total bilirubin (0.17 mg/dL), indicating hepatic injury. MCLME treatment, on the other hand, resulted in a considerable decrease in these biochemical indicators, with SGOT, SGPT, ALP, and bilirubin levels dropping to 54.5 U/L, 15.83 U/L, 134.98 IU/L, and 0.105 mg/dL, respectively. Similarly, the conventional silymarin‐treated group showed significant improvement in these parameters, demonstrating the hepatoprotective effect.

### 3.6 Assessment of Pro‐Inflammatory Cytokine IL‐6 in Serum

**FIGURE 5:**
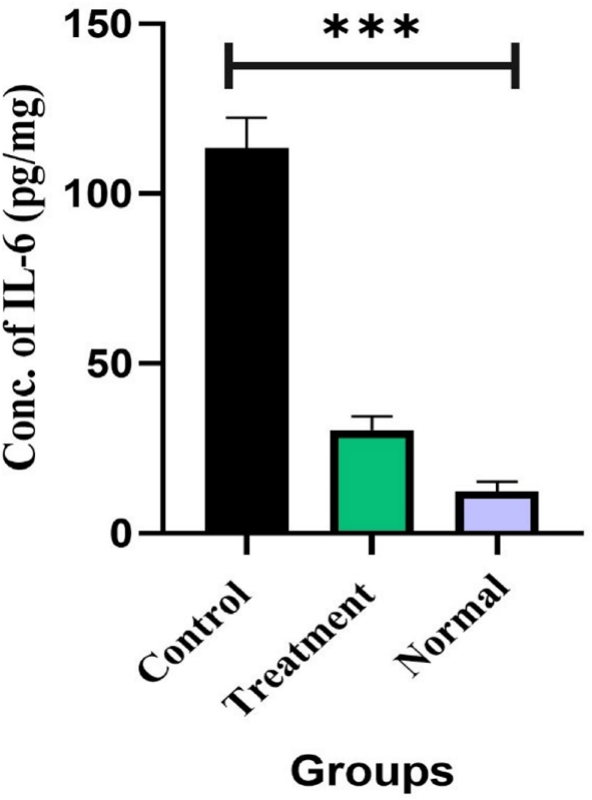
Measurement of pro‐inflammatory cytokine IL‐6 in the serum of the tested mice. MCLME showed a notable reduction of serum IL‐6 levels in the treated group (30.33 ± 4.09) in contrast of the paracetamol intoxicated group (113.46 ± 8.80). Values are expressed as mean ± SEM (*n* = 6 per group). Statistical significance is indicated as ***p < 0.001 compared to the paracetamol intoxicated group.

Serum levels of the pro‐inflammatory cytokine IL‐6 were measured in the experimental mouse groups. Paracetamol‐intoxicated mice exhibited a marked elevation of IL‐6 (113.46 ± 8.80) compared to the normal group (12.29 ± 2.83), confirming that paracetamol induces severe inflammation. Interestingly, treatment with MCLME significantly reduced IL‐6 levels to 30.33 ± 4.09 (*p* < 0.0001), demonstrating its strong anti‐inflammatory efficacy (Figure 5).

### 3.7 Evaluation of the Histopathology of the Liver

**FIGURE 6:**
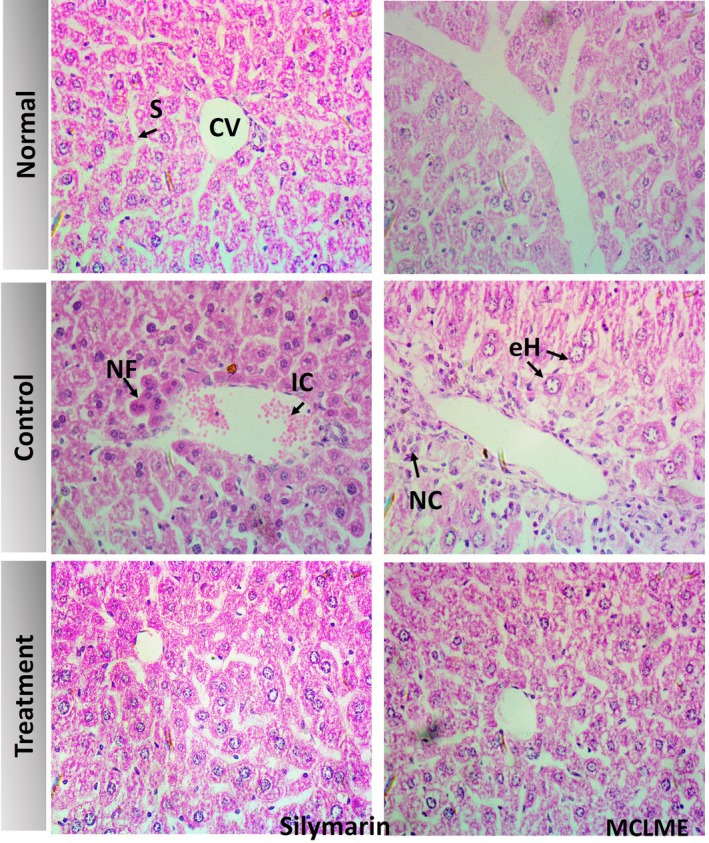
Histopathological examination of liver tissues from experimental groups. Liver sections were stained with hematoxylin and eosin (H&E) and observed under a light microscope at 400× magnification. Scale bar: 40 μm. The normal group showed normal architecture; the paracetamol‐treated group exhibited necrosis, vein dilation, and inflammation. Meanwhile, the Silymarin‐ and MCLME‐treated groups showed improved structure with reduced necrosis and inflammation, indicating hepatoprotection. CV, central vein; eH, enlarged hepatocytes; IC, inflammatory cells; NC, necrotic cells; NF, nuclear fragmentation; S, sinusoid.

The histological investigation indicated major variations between the experimental groups. The normal group (Group 1) had conventional liver architecture, with well‐defined hepatic lobules. Hepatocytes were distributed in radiating cords from the central vein, with distinct sinusoids, consistent cell shape, round nuclei, intact cytoplasm, and no evidence of inflammation In contrast, the paracetamol‐treated group (Group 2) had substantial hepatic injury, including extensive centrilobular necrosis, dilatation of the central vein, and significant inflammatory cell infiltration. Notably, the groups receiving Silymarin (50 mg/kg body weight/day, p.o.) and MCLME (200 mg/kg body weight/day, p.o.) showed significant histological improvements. Liver slices from these groups showed lobular structural repair, decreased necrosis, and reduced inflammatory infiltration, indicating hepatoprotective benefits (Figure 6).

## 4 Discussion

The liver is the largest and most significant organ in the human body for detoxification and drug processing. Hepatocytes are therefore vulnerable to harmful substances as they carry out our bodies' detoxification processes (Kabir et al. 2024). Hepatitis, liver cirrhosis, and other potentially fatal liver illnesses can result from the repeated liver damage caused by a variety of drugs and chemicals. Experimental animals have been reported to be susceptible to liver damage when exposed to paracetamol (Mossanen and Tacke 2015). In this study, MCLME was evaluated for its potential hepatoprotective effects against paracetamol‐induced liver damage in mice to determine the correlation between antioxidant and hepatoprotective activity.

Generally, serum transaminases, including SGOT, SGPT, and ALP, are typically employed as significant markers of hepatic injury (Ajiboye et al. 2015). Among these, SGPT is a crucial cytosolic enzyme that is more liver‐specific, whereas SGOT is primarily found in the hepatic mitochondria along with other organs such as the heart, kidneys, muscles, and brain. Thus, a higher level of SGOT in the blood indicates that cells in one or more of these organs have been damaged or injured; therefore, it is a less‐liver‐specific marker for liver disease compared to that of SGPT (Ndrepepa 2021). Obstruction as well as inflammation of the bile duct has been shown to elevate plasma ALP activity. Increased blood levels of ALP are mainly attributed to the release of transaminases from hepatocytes into the bloodstream, signifying liver injury or dysfunction (Pujari and Bandawane 2021). Bilirubin is a critical clinical indicator for the assessment of liver function, particularly in the context of hepatocellular injury and bile excretion. Hyperbilirubinemia, or elevated bilirubin levels, frequently indicates liver dysfunction, as damaged hepatocytes release bilirubin during the degradation of heme proteins (Lei et al. 2023). Our research demonstrated that mice administered paracetamol experienced a substantial increase (*p* < 0.001) in serum levels of SGPT, SGOT, and ALP when compared with the control group. These liver enzymes are frequently elevated in response to liver cell injury, which indicates impaired liver function and hepatocellular leakage. This is consistent with the hepatotoxic potential of paracetamol, which has been previously reported by Balamurugan (2007) and Kanchana and Sadiq (2011). However, the co‐treatment of MCLME and Silymarin resulted in a significant decrease in these elevated enzyme levels, suggesting that both agents have hepatoprotective activity. MCLME may assist in the stabilization of the membranes of hepatocytes and the restoration of liver function, as indicated by the decrease in liver enzymes. Similarly, the serum total bilirubin level in the paracetamol‐intoxicated group exhibits a significant change compared to the standard, treated, and normal animals. This could imply that the damages were sufficient to cause a significant increment in serum transaminases leakage along with impaired bilirubin excretion and accumulation, which could be nullified by the supplementation of the extract. Importantly, there is no significant difference between standard (Silymarin) and MCLME in the examined parameters implying that the hepatoprotective effect of the extract is quite comparable to the standard drug. Therefore, MCLME could be a potential resource for effective hepatoprotective agents.

The evaluation of liver histopathology is an important step in determining the therapeutic potential of hepatoprotective drugs because it offers direct visual evidence of tissue injury and recovery. Histological changes provide excellent information regarding the amount of hepatocellular injury and inflammation, and they serve as an important complement to biochemical enzyme tests (Gasmi and Kleiner 2020). In this study, paracetamol caused significant histological damage, including necrosis, inflammatory infiltration, and architectural disruption of hepatocytes. However, treatment with MCLME and Silymarin considerably improved these pathological alterations. The restoration of hepatocyte structure, reduction in inflammatory cell infiltration, and reorganization of hepatic lobules suggest that both therapies had significant hepatoprotective benefits. The treated groups' near‐normal histological appearance corroborates the biochemical data, which revealed lower levels of liver enzymes such as SGPT, SGOT, and ALP. This link indicates that MCLME, like the usual hepatoprotective drug Silymarin, not only reduces hepatic enzyme leakage but also helps to preserve or restore liver tissue integrity. These findings are similar to prior research, such as Balamurugan (2007), who found that antioxidant‐rich plant extracts can normalize serum indicators and repair liver tissue damage after toxic assaults. It is assumed that MCLME's hepatoprotective impact is likely due to its antioxidant and anti‐inflammatory characteristics of the extract.

N‐acetyl‐p‐benzoquinone imine (NAPQI) is a toxic metabolite produced from paracetamol metabolism; it is pivotal in generating oxidative stress by depleting intracellular antioxidants, especially glutathione (GSH). This depletion disturbs the equilibrium between free radical formation and the antioxidant defense mechanism, resulting in oxidative stress that undermines cellular homeostasis and contributes to hepatocellular damage (Crosas‐Molist and Fabregat 2015). The current study reveals that the group treated with paracetamol had significantly lower GSH levels, underscoring the heightened generation of ROS, which undermines the liver's inherent antioxidant capability and leads to tissue damage. Treatment with MCLME and the conventional medication Silymarin significantly reduced the paracetamol‐induced depletion of GSH. This restoration of GSH indicates that the extract has strong antioxidant qualities that can improve the liver's defense mechanisms and reduce oxidative damage.

The abundance of bioactive phytochemicals found in medicinal plants is primarily responsible for their therapeutic potential. The phytochemical analysis of *M. canadensis* leaves conducted for our study revealed the presence of a number of important compounds, such as terpenoids, alkaloids, flavonoids, and saponins. The elevated concentrations of total phenolic and flavonoid compounds are likely factors contributing to the plant's considerable antioxidant capacity. These substances are recognized for their hepatoprotective benefits via many mechanisms, including free radical scavenging, anti‐inflammatory activity, and modulation of hepatic enzyme function (Saha et al. 2019). Flavonoids and phenolic chemicals included in *M. canadensis* have demonstrated the ability to mitigate the processes involved in hepatic damage. They can inhibit the synthesis of proinflammatory cytokines (Kim et al. 2011) and augment the expression of endogenous antioxidant enzymes, including superoxide dismutase (SOD), catalase (CAT), and glutathione peroxidase (GPx). Additionally, they facilitate the restoration of intracellular glutathione (GSH), an essential component in the detoxification of NAPQI and ensuring that redox equilibrium is preserved (Kruk et al. 2022). According to previous reports, the development of NAPQI protein adducts causes tissue damage and inflammation by inhibiting anti‐inflammatory mechanisms in experimental animals. It also increases the production of pro‐inflammatory cytokine IL‐6, which in turn is associated with liver injury through the activation of the trans‐signaling pathway (Dkhil et al. 2019). Interestingly, administration of MCLME led to a significant reduction in serum IL‐6 levels as previously reported (Nithiyanandam and Prince 2023). The findings indicate that the hepatoprotective benefits of MCLME identified in this study are likely facilitated by its antioxidant and anti‐inflammatory phytochemicals, which aid in restoring hepatic integrity and functions.

Additionally, the antioxidant capacity of the methanolic extract of *M. canadensis* leaves (MCLME) has been evaluated through the performance of DPPH and ABTS radical scavenging assays, along with the lipid peroxidation test. The DPPH assay evaluates the capacity of antioxidants to donate hydrogen atoms, thereby neutralizing free radicals (Constantin et al. 1990). Importantly, the inhibition of lipid peroxidation processes is frequently linked to the scavenging of DPPH radicals (Ratty et al. 1988; Rekka and Kourounakis 1991). In both in vivo and in vitro models, paracetamol‐induced hepatotoxicity is well documented to be a result of lipid peroxidation, a critical event in oxidative stress (Wang et al. 2017). The present study exhibited a remarkable percentage of lipid peroxidation inhibition in our test sample—MCLME. In DPPH and ABTS assays, MCLME demonstrated significant radical scavenging activity, as evidenced by a relatively low IC_50_ value, which suggests its ability to effectively neutralize free radicals. This study is supported by previous findings where the *Mentha* spp. has been documented to possess high antioxidant capacity (Tafrihi et al. 2021). These results indicate that MCLME can effectively mitigate oxidative stress by neutralizing ROS, which in turn reduces lipid peroxidation and safeguards cellular components, including membranes, proteins, and DNA, from oxidative damage. Moreover, MCLME showed potent human RBC membrane stabilizing capacity, suggesting the ability to protect the lysosomal membrane to inhibit inflammation (Chowdhury et al. 2013). This result remains consistent with the inhibition of protein denaturation assay, where MCLME elicited a noteworthy result that confirms the protection of cellular components from oxidative damage. The therapeutic potential of MCLME in mitigating drug‐induced liver toxicity is supported by the hepatoprotective effects demonstrated in vivo, which are likely influenced by the antioxidant properties observed in vitro.

## 5 Conclusion and Future Perspectives

In summary, the hepatoprotective activity found in the MCLME can be attributed principally to its powerful free radical‐scavenging and antioxidant abilities. These benefits are most likely due to the presence of bioactive phytochemicals, particularly flavonoids and phenolic compounds, which have been thoroughly studied for their functions in reducing oxidative stress and moderating inflammatory responses. These chemicals, by neutralizing ROS, may assist with preserving cellular integrity, inhibit lipid peroxidation, and aid in the restoration of liver function after the toxic insult. Despite these hopeful results, more research is needed to completely understand the defensive mechanism involved. Bioassay‐guided fractionation, as well as advanced chromatographic and spectroscopic techniques, should be used to extract, identify, and characterize the individual flavonoids, phenolics, and other active ingredients that cause the observed effects. Furthermore, mechanistic investigations, such as gene expression monitoring of antioxidant enzymes and pro‐inflammatory markers, would provide a better understanding of how these substances interact with cellular pathways to confer hepatoprotective effects. Therefore, further studies will be crucial in establishing *M. canadensis'* therapeutic potential and aiding the development of plant‐based therapies for liver problems.

## Author Contributions

**Asma Ul Husna Biswas:** formal analysis (equal), investigation (lead), writing – original draft (lead). **Tasnima Kamal:** data curation (equal), visualization (equal), writing – review and editing (supporting). **Azmin Akter:** formal analysis (supporting), investigation (supporting). **Sharmin Akter:** formal analysis (supporting), methodology (supporting). **Azadur Rahman Bhuiyan:** data curation (supporting), formal analysis (supporting), methodology (supporting). **Zinnat Ara Moni:** data curation (supporting), visualization (supporting). **Abdul Auwal:** project administration (supporting), visualization (supporting). **Nitai Roy:** writing – review and editing (equal). **Shakhawoat Hossain:** supervision (supporting), writing – review and editing (supporting). **Farhadul Islam:** conceptualization (equal), writing – review and editing (equal).

## Funding

This work was supported by grants from the Dean of Science, Rajshahi University, Rajshahi‐6205, Bangladesh (Grant No: 2002.5/52/RU/Science‐35/2023‐2024).

## Ethics Statement

The Institutional Animal, Medical Ethics, Biosafety, and Biosecurity Committee (IAMEBBC) at the Institute of Biological Sciences, University of Rajshahi, Bangladesh (128/320‐IAMEBBC/IBSc), sanctioned the utilization of animals for this research.

## Conflicts of Interest

The authors declare no conflicts of interest.

## Data Availability

The data that support the findings of this study are available on request from the corresponding author.
